# CD34^+^CD10^+^CD19^−^ Cells in Patients with Unhealthy Alcohol Use Stimulate the M2b Monocyte Polarization

**DOI:** 10.3390/cells11172703

**Published:** 2022-08-30

**Authors:** Akira Asai, Yusuke Tsuchimoto, Hideko Ohama, Hiroki Nishikawa, Ashok Chopra, Kazuhide Higuchi

**Affiliations:** 1 2nd Department of Internal Medicine, Osaka Medical and Pharmaceutical University, Takatsuki 569-8686, Japan; 2 Department of Microbiology and Immunology, The University of Texas Medical Branch, Galveston, TX 77555, USA

**Keywords:** patients with unhealthy alcohol use 2, CD34^+^CD10^+^CD19^−^ cells 3, M2b monocytes 4, HMGB1

## Abstract

M2b monocytes commonly isolated from patients with unhealthy alcohol use (Alc) have been described as cells that make the host susceptible to opportunistic infections. CD34^+^CD10^+^CD19^−^ cells are multilineage progenitors of CD19^+^ cells, and we show that the effect of these cells from the peripheral blood on M2b monocyte polarization differed between healthy donors and Alc in this study. In healthy donors, these cells consistently differentiated into high-mobility group box-1 (HMGB1)-nonproducing cells (CD19^+^ cells) in response to retinoic acid (RA). However, owing to the lack of expression of RA receptor (RAR), these cells from Alc failed to differentiate into CD19^+^ cells under the same RA stimulation. Conditioned medium (CM) of these cells from Alc induced the polarization of M2b monocytes, which increases the susceptibility of hosts to opportunistic infections in Alc. When the alcoholic individuals were subjected to 2 weeks of abstinence from alcohol, these cells from Alc recovered their RAR expression and differentiated into CD19^+^ cells. Moreover, the CM of these cells from Alc after abstinence lost its ability to induce M2b monocyte polarization. These results indicate that these cells from Alc have different properties from those of healthy donors. In Alc, these cells without RAR stimulate M2b monocyte polarization through the production of HMGB1.

## 1. Introduction

Alcohol use usually has a variety of negative health outcomes, including morbidity, mortality, and disability. Every year, 3 million deaths result from alcohol use, representing 5.3% of all deaths globally [1]. Many hospitalized people with unhealthy alcohol use are diagnosed with infectious complications [2] and the majority of the causative pathogens for infections in people with unhealthy alcohol use are the microbes normally found in the upper and lower intestines [3]. Because such infections do not usually develop in healthy individuals, certain immune dysfunctions related to alcohol are considered to be the underlying causes of the increased susceptibility of people with unhealthy alcohol use to these infections. In fact, various host antibacterial immune functions are strongly influenced by unhealthy alcohol use [4,5,6,7,8,9,10,11,12,13,14]. Decreased immune function associated with alcohol use includes granulopoiesis, tissue recruitment of neutrophils, Toll-like receptor responsiveness of macrophages (Mφ) [7], decreased IL-12 production [8], and increased Th2 responses [14]. We previously showed that gut bacteria-associated sepsis is controlled by bactericidal M1Mφ (IL-10^–^IL-12^+^CCL1^–^ Mφ), which results in infections [3]. However, M1Mφ is not easily generated in people with unhealthy alcohol use because M2bMφ (IL-10^+^CCL1^+^ Mφ), predominantly generated in people with unhealthy alcohol use, inhibits the conversion of M1Mφ from monocytes [15]. However, it remains unclear as to why M2bMφ/monocytes are generated in individuals with chronic alcohol consumption.

CD34^+^ cells are multipotent somatic cells that can differentiate into various cell types and support regeneration and restoration in vivo [16]. They affect immune regulatory functions in both adaptive and innate immunity [17,18]. The models of human B-lineage development suggest a single hierarchical development pathway from CD34^+^ stem cells to B cells [19]. Ordered stages in the current human model are termed the “common lymphoid progenitor (CLP)” and “Pre/Pro-B,” “Progenitor-B,” and “Precursor-B” subsets that follow it (i.e., stem cell → CLP → Pre/Pro B cells → Pro-B cells → Pre-B cells → B cells). Pre/Pro B cells, which retain the expression of CD34 and CD10 but not of CD19, constitute the first B-lineage stage, followed by Pro-B cells.

Vitamin A has a wide spectrum of actions in a developmental stage-specific manner. Most of the actions of vitamin A are exerted through nuclear receptor-mediated gene expression [20]. The active form of vitamin A is identified as RA, and it specifically binds to the nuclear receptors RAR and retinoic X receptors, which belong to a gene superfamily of nuclear steroid/thyroid hormone receptors, and act as ligand-inducible transcription factors [21]. Although many transcriptional factors are involved in cell development and polarization, we focused on RA.

In this study, we investigated the properties of CD34^+^CD10^+^CD19^−^ cells in the peripheral blood of people with unhealthy alcohol use and the effect of these cells on M2bMφ/monocyte polarization.

## 2. Patients and Methods

### 2.1. Ethics Statement

All procedures performed in this study were in accordance with the ethical standards of the institution, ethical guidelines for medical and human subjects in Japan, and the 1964 Helsinki Declaration and its later amendments. The study was approved by the Institutional Review Board of Osaka Medical and Pharmaceutical University (IRB approval number: 2125-1). Written informed consent for blood sampling was obtained from all the patients and all healthy donors.

### 2.2. Patients and Healthy Donors

The study was carried out on 18 patients (13 men and 5 women) who were diagnosed as people with unhealthy alcohol use at Osaka Medical College from August 2016 to December 2018. We enrolled patients who presented to our hospital for the first time and were diagnosed with severe alcohol use disorder, according to The Diagnostic and Statistical MI of Mental Disorders, Fifth Edition (DSM-5) from the American Psychiatric Association. All patients had no active infections. We collected patient information using Alcohol Use Disorders Identification Test (AUDIT) and assessed the patient’s alcohol intake through interviews with the medical staff at every time of hospital visit. Alcohol abstinence was defined as abstinence from alcohol for 2 weeks. The patients, on average, consumed 124.5 ± 63.6 g/day of ethanol for 8 to 49 years (26.7 ± 13.3 years) (Table 1). Patients who had any malignancy, heart failure, chronic kidney disease, liver cirrhosis, or infection were excluded from this study. Eligible patients provided written informed consent to participate in the study, and the protocol was approved by the ethics committee of the hospital. People who were diagnosed as healthy by a complete medical checkup within one year were enrolled as healthy donors (HD). As HD, 11 non-alcoholic individuals (9 men and 2 women; mean age, 36.1 years) were enrolled. None of the HD consumed more than 10 g/day of ethanol.

### 2.3. Bacterium, Reagents, and Media

*Enterococcus faecalis* (29212 strain) was purchased from the American Type Culture Collection (Manassas, VA, USA). In accordance with the guidelines for assuring the quality of medical microbiological culture media, bacteria were grown in trypticase soy broth for 18 h at 37 °C under aerobic conditions before being used in infection experiments. HMGB1 ELISA kit was purchased from Qarigo Biolaboratories (Taiwan), and RA ELISA kit was purchased from CUSABIO (Houston, TX, USA). Recombinant CCL1 was obtained from PeproTech (San Diego, CA, USA). Anti-CCL1 mAbs for ELISA were obtained from R&D Systems (San Diego, CA, USA). IL-12 and IL-10 ELISA kits were purchased from Biolegend (San Diego, CA, USA). For reverse transcription PCR, RNeasy Mini Kit, QuantiTect Reverse Transcription Kit, miRCURY LNA miRNA PCR Starter Kit, miR-27a primer, and miR-222 primer were purchased from QIAGEN KK (Tokyo, Japan). For flow cytometry, antibodies directed against CD3, CD10, CD14, CD19, CD34, CD56, IL-10, and RA receptors were purchased from Biolegend and United States Biological (Salem, MA, USA). Isotype control mAbs, Cytofix/CytopermTM solution, anti-CD14 magnetic particles, and IMagTM buffer were purchased from BD Biosciences (San Jose, CA, USA). The Lineage cell depletion kit was purchased from Miltenyi Biotech (Tokyo, Japan). For cell cultivation, RPMI-1640 medium supplemented with 10% heat-inactivated FBS, 2 mM L-glutamine, and antibiotics (100 U/mL penicillin and 100 μg/mL streptomycin) (complete medium) was used.

### 2.4. Isolation of CD14^+^ and CD34^+^CD10^+^CD19^−^ Cells

Peripheral blood mononuclear cells (PBMCs) of HD, patients with unhealthy alcohol use (Alc), and abstained patients were isolated from 20 mL of heparinized whole blood by Ficoll-Hypaque density gradient centrifugation. In all experiments, we used freshly isolated PBMCs. PBMCs are first labeled with a cocktail of biotin-conjugated antibodies against lineage-specific antigens (CD2, CD3, CD11b, CD14, CD15, CD16, CD19, CD56, CD123, and CD235a (Glycophorin A)). These cells were subsequently magnetically labeled with Anti-Biotin MicroBeads. Lineage-negative cells were obtained by depletion of the magnetically labeled cells. When these obtained cells were stained by anti-CD19 mAb, 96% of them were shown to be CD19^−^ cells. Then, these cells were stained for CD34 and CD10 by anti-CD34 and anti-CD10 mAbs, and CD34^+^CD10^+^CD19^−^ cells were sorted from the stained cells using FACS Aria. Finally, 3–5 *×* 10^4^ cells of CD34^+^CD10^+^CD19^−^ cells were obtained from 20 mL of peripheral blood of HD, Alc and abstained Alc.

### 2.5. Experiments Using CD14^+^ and CD34^+^CD10^+^CD19^−^ Cells

CD34^+^CD10^+^CD19^−^ cells were stained with APC-conjugated anti-RA receptor mAb, and the percentage of RA receptor-expressing cells was analyzed by flow cytometry. In some experiments, CD34^+^CD10^+^CD19^−^ cells (1 × 10^6^ cells/mL) were cultured with or without different doses of RA for 24 h. Cells differentiated into CD3^+^, CD14^+^, CD19^+^, or CD56^+^ cells from the CD34^+^CD10^+^CD19^−^ cell preparation were analyzed by flow cytometry. To obtain 24 h-conditioned medium (CM) of CD34^+^CD10^+^CD19^−^ cells (CD10^+^CD19^−^CM), these cells (1 × 10^6^ cells/mL) were cultured with 3 ng/mL RA for 24 h. After washing, the cell preparations were recultured for an additional 24 h and conditioned medium (CM) was obtained. CM was assayed for high mobility group box-1 (HMGB1) by ELISA and used to determine its ability to polarize monocytes (CD14^+^ cells).

### 2.6. Characterization of CD14^+^ Cells

Peripheral blood CD14^+^ cells from healthy donors (1 × 10^6^ cells/mL) were cultured in a medium supplemented with 10% (*w/w*) of CD10^+^CD19^−^CM. As a control, CD14^+^ cells from HD were cultured without any stimulation. Twenty-four hours after cultivation, the cells were washed and recultured for 24 h. The harvested culture fluids were assayed for CCL1 using ELISA. The minimum detection limit for the abovementioned cytokines in our assay system was 4–13 pg/mL. The obtained CD14^+^ cells were permeabilized with Cytofix/Cytoperm solution at 4 °C for 20 min and then washed with a Perm/Wash solution. The cells were stained with anti-IL-10 mAb and analyzed for IL-10 expressing cells using FACS Canto and FlowJo software (BD Biosciences). IL-10 is a biomarker of M2 monocytes/M*φ* and CCL1 is a biomarker of M2b monocytes/M*φ*. M2b monocytes were detected as IL-10^+^CCL1^+^CD14^+^ cells.

To determine the bactericidal activity of monocytes, CD14^+^ cells (1 × 10^6^ cells/mL) suspended in antibiotic-free RPMI-1640 medium supplemented with 10% FBS were exposed to 2.5 × 10^5^ CFU/mL of Enterococcus faecalis (*E. faecalis*). For comparison, the pathogen was incubated alone under the same conditions in the “compared group.” Three hours after incubation, samples were lysed in 0.1% Triton-X (Sigma), and serial 10-fold dilutions of lysates were plated on tryptic soy agar. The number of colonies was counted after incubation for 24 h at 37 °C. The following formula was applied to the results: bactericidal activity = (1–test group CFU/compared group CFU) × 100.

### 2.7. Infection Mouse Model

Seven- to ten-week-old pathogen-free, male NOD.Cg-Prkc^scid^Il12rg^tm1wjl^/Szj (NSG) mice were purchased from the Jackson Laboratory (Tokyo, Japan). NSG mice lack functional T, B, and NK cells [22,23,24], and are carriers of macrophages with defective phagocytosis, digestion, and antigen presentation [3,25]. All NSG mice were exposed to whole-body irradiation (4 Gy) to deplete neutrophils (X-irradiated NSG mice) [15]. Neutrophils did not recover in these mice for 4 weeks after irradiation. Monocytes (1 × 10^6^ cells/mouse) were inoculated into X-irradiated NSG mice. Two days after cell inoculation, the mice were infected with E. faecalis (2 × 10^6^ CFU/mouse, IV).

### 2.8. Statistical Analyses

Statistical analyses were performed using the JMP Pro software ver. 14 (Tokyo, Japan). Quantitative values are expressed as means. Differences in quantitative values between the two groups were analyzed using the Mann-Whitney U test. Differences in the ratio between the two groups were analyzed using Fisher’s exact test. For survival analysis, the Kaplan-Meier method was used to analyze the overall survival, and the log-rank test was used for comparisons. Results were considered significant if the *p*-value was <0.05. The results were corrected using the Holm method if multiple comparisons were made during the analysis.

## 3. Results

### 3.1. CD34^+^CD10^+^CD19^−^ Cells in the Peripheral Blood from Patients with Unhealthy Alcohol Use

**Figure 1:**
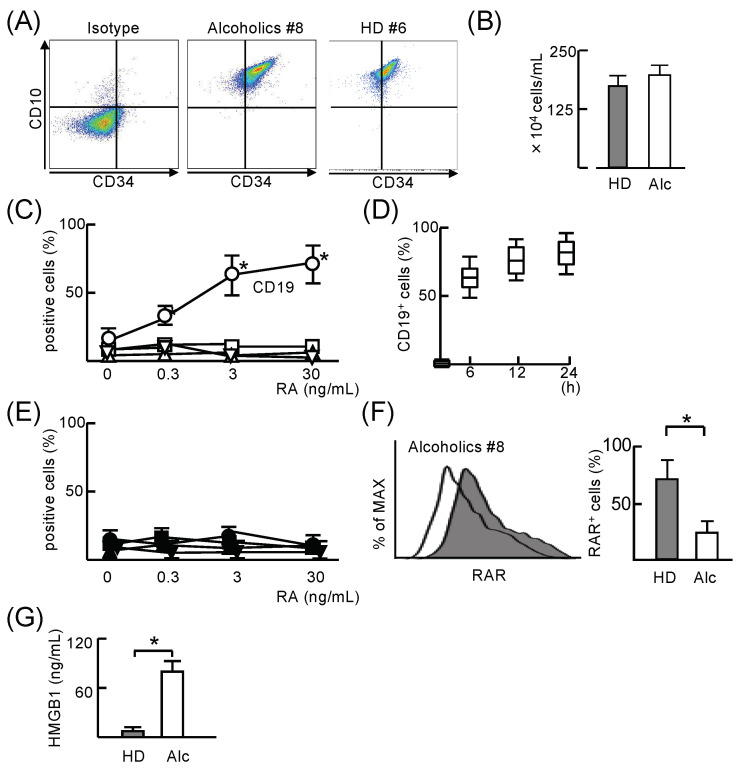
CD34^+^CD10^+^CD19^−^ cells in the peripheral blood of healthy donors and patients with unhealthy alcohol use. (**A**) CD34^+^CD10^+^CD19^−^ cells were isolated from lineage-negative cells by flow cytometry. (**B**) The number of CD34^+^CD10^+^CD19^−^ cells in the peripheral blood from patients with unhealthy alcohol use (Alc; #1–11) and healthy donors (HD; #1–11). (**C**–**E**) CD34^+^CD10^+^CD19^−^ cells from healthy donors (**C**) and Alc (**E**) were cultured with or without different doses of retinoic acid (RA) for 24 h. These cells were stained with mAbs directed against CD3 (triangle), CD14 (square), CD19 (circle), and CD56 (inverted triangle) and analyzed by flow cytometry. (**D**) The same cell preparation from HD was stimulated with 3 ng/mL of RA for 1 to 24 h. These cells were stained with CD19 mAbs. (**F**) CD34^+^CD10^+^CD19^−^ cells from Alc (#8–16) and HD (#5–11) were stained with APC-conjugated anti-retinoic acid receptor (RAR) mAb and analyzed by flow cytometry. (**G**) Production of HMGB1 by CD34^+^CD10^+^CD19^−^ cells from Alc (#13–18) and HD (#5–10) stimulated with 3 ng/mL RA, as assessed by ELISA. Data are presented as mean ± SD. * *p* < 0.05.

Lineage-negative cells which were negative for CD2, CD3, CD11b, CD14, CD15, CD16, CD19, CD56, CD123, and CD235a, were isolated from PBMC of patients with unhealthy alcohol use (Alc) and health donors (HD) using a lineage-negative isolation kit. CD34^+^CD10^+^CD19^−^ cells were sorted from lineage-negative cells by flow cytometry (Figure 1A). There was no difference in the number of CD34^+^CD10^+^CD19^−^ cells in the peripheral blood of HD and Alc (Figure 1B). Retinoic acid, metabolized from vitamin A, is essential for both normal embryonic development and the maintenance of differentiation in adult organisms [20]. Therefore, the RA-responsiveness of CD34^+^CD10^+^CD19^−^ cells from Alc was compared to that of the same cell preparation from HD. When CD34^+^CD10^+^CD19^−^ cells from HD were stimulated with RA, they differentiated into CD19^+^ cells but not CD3^+^, CD14^+^, or CD56^+^ cells (Figure 1C). The maximum differentiation (90%) of CD34^+^CD10^+^CD19^−^ cells into CD19^+^ cells was observed 1 h after RA stimulation (Figure 1D). However, CD34^+^CD10^+^CD19^−^ cells from Alc did not differentiate into CD3, CD14, CD19, or CD56 cells even when they were stimulated with RA (Figure 1E). The majority of CD34^+^CD10^+^CD19^−^ cells from HD expressed the retinoic acid receptor (RAR), whereas the RAR expression in the same cell preparation from Alc was significantly reduced (Figure 1F). When CD34^+^CD10^+^CD19^−^ cells from Alc were stimulated with RA for 24 h, they produced 97 ± 3.5 ng/mL HMGB1. However, these cells from HD did not produce HMGB1 (Figure 1G). These results indicate that CD34^+^CD10^+^CD19^−^ cells from Alc, unlike those from HD, produced HMGB1 and lacked the ability to differentiate into CD19 cells in response to RA.

### 3.2. Conditioning Medium of CD34^+^CD10^+^CD19^−^ Cells from Patients with Unhealthy Alcohol Use (CD10^+^CD19^−^ CM-Alc) Induces M2b Monocyte Polarization

**Figure 2:**
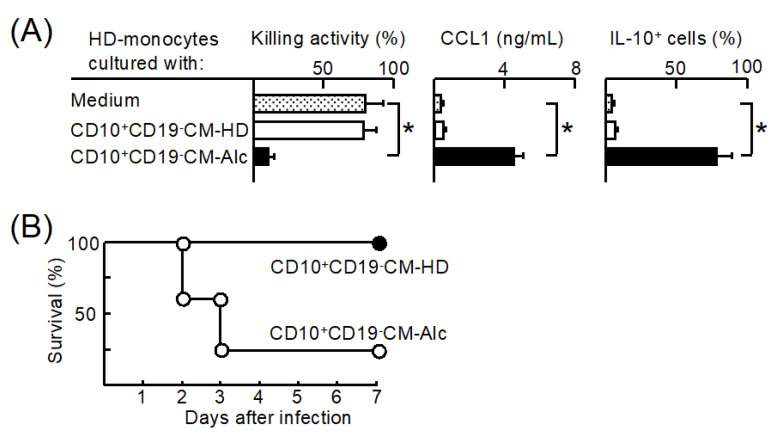
Influence of conditioning medium (CM) of CD34^+^CD10^+^CD19^−^ cells from patients with unhealthy alcohol use (CD10^+^CD19^−^CM-Alc) on M2b monocyte polarization. (**A**) CD34^+^CD10^+^CD19^−^ cells (1 × 10^6^ cells/mL) from Alc (#4–11) and HD (#1–8) were cultured with 3 ng/mL RA for 24 h. After washing, these cell preparations were recultured for an additional 24 h and each CM was obtained. Each CM was mixed with healthy donor-derived monocytes (HD monocytes) for 24 h. As a control, the same monocytes were cultured without any stimulation. The obtained cells were tested for the properties of M2b monocytes. (**B**) Monocytes from healthy donors (#1–6) were stimulated with 10% syngeneic CD10^+^CD19^−^CM-HD for 24 h. HD monocytes were stimulated with CD10^+^CD19^−^CM-Alc (#4–9) for 24 h. After washing, the obtained monocytes were inoculated into X-irradiated NSG mice, which were then intravenously infected with *E. faecalis* (2 × 10^6^ CFU/mice). Data are presented as mean ± SD. * *p* < 0.05.

We previously reported that antibacterial resistance in Alc is inhibited by M2b monocyte polarization in the peripheral blood [3]. Therefore, we examined the influence of CD34^+^CD10^+^CD19^−^ cells from Alc on M2b monocyte polarization. Twenty-four hour-conditioning medium (CM) of CD34^+^CD10^+^CD19^−^ cells stimulated with RA (CD10^+^CD19^−^CM) was added to normal CD14^+^ cells (HD monocytes). The obtained cells were then tested for the biomarkers of M2bMφ/monocytes. M2bMφ/monocytes have been shown to be IL-10^+^CCL1^+^cells with a reduced capacity to kill pathogens [26]. HD monocytes cultured with CD10^+^CD19^−^ CM from healthy donors (CD10^+^CD19^−^CM-HD) were observed to be IL-10^−^CCL1^−^cells with killing activity against *E. faecalis*. However, HD monocytes cultured with CD10^+^CD19^−^CM from Alc (CD10^+^CD19^−^CM-Alc) were IL-10^+^CCL1^+^ cells lacking bactericidal activity (Figure 2A). Most of the X-irradiated NSG mice inoculated with HD monocytes stimulated with CD10^+^CD19^−^CM-Alc died after *E. faecalis* infection. However, the same mice inoculated with HD monocytes stimulated with CD10^+^CD19^−^CM-HD survived after infection (Figure 2B). These results indicate that CM from CD34^+^CD10^+^CD19^−^ cells from Alc stimulated with RA has the ability to stimulate M2b monocyte polarization. However, CM of CD34^+^CD10^+^CD19^−^ cells from HD stimulated with RA did not polarize HD monocytes to M2b monocytes.

### 3.3. Effect of HMGB1 on M2b Monocyte Polarization

**Figure 3:**
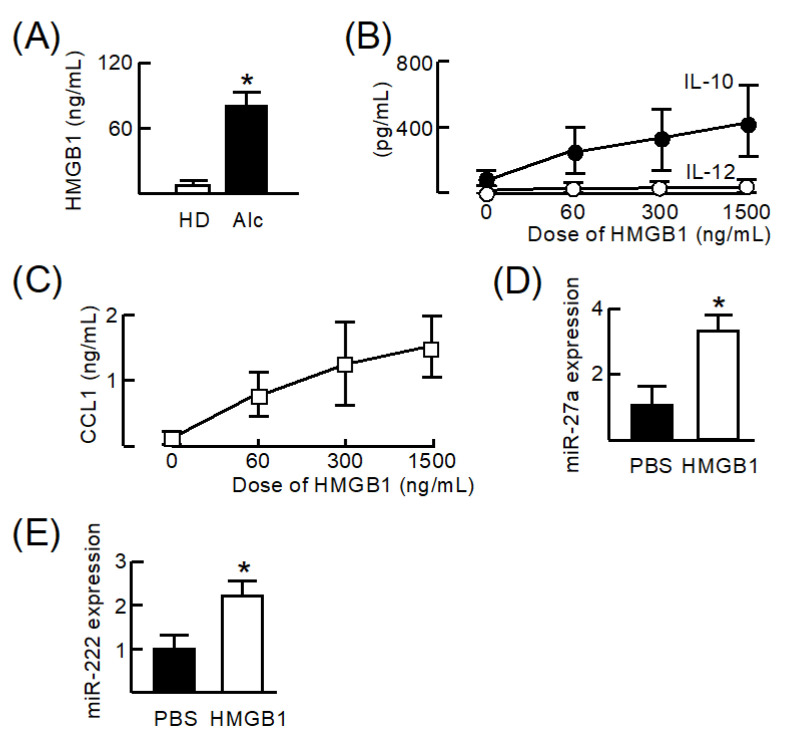
Effect of HMGB1 on M2b monocyte polarization from healthy donor-derived monocytes. (**A**) CD34^+^CD10^+^CD19^−^ cells from healthy donors or patients with unhealthy alcohol use were stimulated with 3 ng/mL retinoic acid (RA) for 24 h. HMGB1 production from these cells was assayed by ELISA. (**B**,**C**) Monocytes isolated from healthy donors were stimulated with different doses of HMGB1. Obtained culture fluids were assayed for IL-12, IL-10, and CCL1 by ELISA. (**D**,**E**) Monocytes isolated from healthy donors were stimulated with 100 ng/mL HMGB1 or cultivated with an equal volume of PBS for 24 h. The expression levels of miR-27a and miR-222 in these cells were assayed by RT-PCR. Data are presented as mean ± SD. * *p* < 0.05.

Production of HMGB1 from CD34^+^CD10^+^CD19^−^ cells was analyzed in vitro. When CD34^+^CD10^+^CD19^−^ cells from HD were stimulated with 3 ng/mL RA for 24 h, these cells did not produce HMGB1. However, the same cells from Alc produced HMGB1 under the stimulation with RA (Figure 3A). Next, to examine the effect of HMGB1 on M2b monocyte polarization, HD monocytes were stimulated with different doses of HMGB1 for 24 h, and the production of some cytokines in culture fluids was assayed by ELISA (Figure 3B,C). When HD monocytes were cultured for 24 h under the stimulation of HMGB1, they produced IL-10 and CCL1, but not IL-12.

Alcohol directly increases the expression of miR-27a and miR-222 in Mφ/monocytes [27] and miR-27a and miR-222 were analyzed by RT-PCR [28]. When HD monocytes were stimulated with 100 ng/mL HMGB1, both miR-27a and miR-222 were expressed in these cells (Figure 3D,E). These results indicate that HMGB1 produced by CD34^+^CD10^+^CD19^−^ cells from Alc may induce M2b monocyte polarization.

### 3.4. Effect of Alcohol Abstinence on CD34^+^CD10^+^CD19^−^ Cells in Patients with Unhealthy Alcohol Use

**Figure 4:**
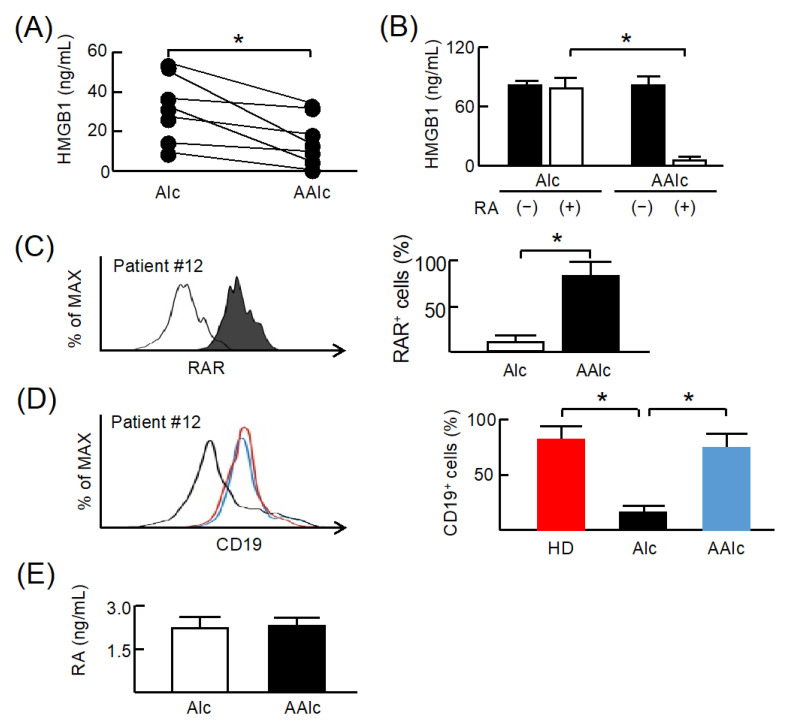
Effect of alcohol abstinence on the expression of retinoic acid receptor (RAR) on CD34^+^CD10^+^CD19^−^ cells from patients with unhealthy alcohol use. (**A**) Serum from patients with unhealthy alcohol use (Alc; #12–18) before and after 2-week alcohol abstinence (AAlc) was assayed for HMGB1 by ELISA. (**B**) CD34^+^CD10^+^CD19^−^ cells from Alc (#12–18) before and after 2-week alcohol abstinence were cultured with or without 3 ng/mL retinoic acid (RA) for 24 h. Obtained culture fluids were assayed for HMGB1 by ELISA. (**C**) CD34^+^CD10^+^CD19^−^ cells from Alc (#12–18) before and after 2-week alcohol abstinence were stained with APC-conjugated RAR mAb and analyzed by flow cytometry. (**D**) After stimulation with 3 ng/mL RA, the obtained cells were stained for CD19 mAb and analyzed by flow cytometry. (**E**) Serum RA from Alc (#12–18) before and after 2-week alcohol abstinence was assayed by ELISA. Data are presented as mean ± SD. * *p* < 0.05.

Abstinence from alcohol has been described as the most important treatment for alcoholism [1]. In a previous study [15], we showed that M2b monocyte polarization in Alc was reduced by short-term abstinence from alcohol. Therefore, we hypothesized that CD34^+^CD10^+^CD19^−^ cells in Alc 2 weeks after alcohol abstinence (AAlc) influences the disappearance of M2b monocytes. Serum obtained from Alc before and 2 weeks after alcohol abstinence were assayed for HMGB1 by ELISA (Figure 4A). Serum HMGB1 levels significantly decreased after alcohol abstinence. In addition, CD34^+^CD10^+^CD19^−^ cells from AAlc did produce HMGB1, but very few, after stimulation with RA (Figure 4B). The expression of RAR on CD34^+^CD10^+^CD19^−^ cells from AAlc was restored to 85% ± 3.5% (Figure 4C). After stimulation with 3 ng/mL RA, most of these cells differentiated into CD19^+^ cells to the levels observed in HD (Figure 4D). In addition, serum RA from Alc 2 weeks after abstinence was almost at the same level as before abstinence (Figure 4E). These results indicate that CD34^+^CD10^+^CD19^−^ cells from Alc recovered RAR expression after alcohol abstinence. These cells were differentiated into CD19^+^ cells after stimulation with RA and the production of HMGB1 from these cells was reduced.

### 3.5. Effect of CD10^+^CD19^−^CM from Alcohol Abstinence on M2b Monocyte Polarization in Patients with Unhealthy Alcohol Use

**Figure 5:**
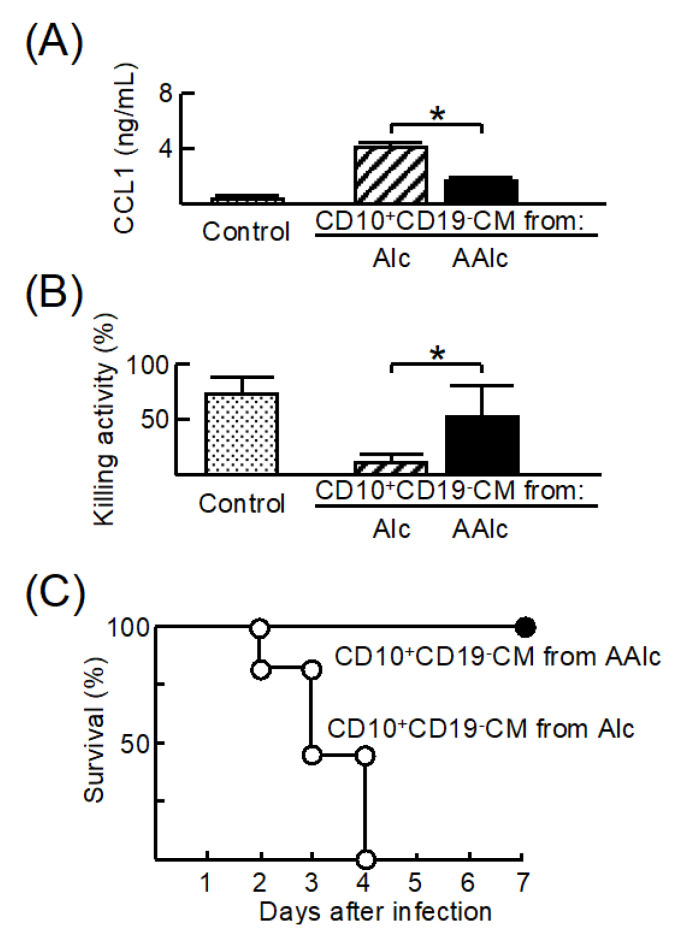
Property of HD monocytes cultured with CD10^+^CD19^−^CM-AAlc. HD monocytes were cultured with CD10^+^CD19^−^CM-Alc or CD10^+^CD19^−^CM-AAlc cells obtained from patients (#12–18). (**A**) CCL1 production and (**B**) bactericidal activity of these cells were examined. (**C**) X-irradiated NOD.Cg-*Prkc^scid^Il12rg^tm1wjl^*/Szj (NSG) mice were inoculated with HD monocytes that were stimulated with CD10^+^CD19^−^CM-Alc or CD10^+^CD19^−^CM-AAlc cells and intravenously infected with *E. faecalis* (2 × 10^6^ CFU/mouse). Data are presented as mean ± SD. * *p* < 0.05.

Next, we analyzed the influence of CD10^+^CD19^−^CM-AAlc on M2b monocyte polarization. CD34^+^CD10^+^CD19^−^ cells were isolated from patients with unhealthy alcohol use before and 2 weeks after alcohol abstinence. Both the groups of cells from syngeneic patients were stimulated with 3 ng/mL RA for 24 h, and CM was obtained (CD10^+^CD19^−^CM-Alc and CD10^+^CD19^−^CM-AAlc). Although HD monocytes stimulated with CD10^+^CD19^−^CM-Alc produced CCL1 and did not have bactericidal activity against *E. faecalis*, monocytes stimulated with CD10^+^CD19^−^CM-Aalc reduced CCL1 production and recovered the bactericidal activity (Figure 5A,B). X-irradiated NSG mice inoculated with HD monocytes stimulated with CD10^+^CD19^−^CM-AAlc survived E. faecalis infection. However, the same mice inoculated with HD monocytes stimulated with CD10^+^CD19^−^CM-Alc died after infection (Figure 5C). These results indicate that the influence of M2b monocyte polarization on CD10^+^CD19^−^CM-Alc was minimal due to alcohol abstinence.

## 4. Discussion

It has long been recognized that patients with unhealthy alcohol use are generally malnourished and can suffer from vitamin A deficiency. However, in this study, serum RA levels in Alc were found to be the same as in HD. When CD34^+^CD10^+^CD19^−^ cells from Alc were stimulated with RA, they were not able to differentiate into other cells because of the deficiency of RAR. Therefore, RA replacement therapy for Alc seems insufficient for the restoration of impaired host antibacterial resistance. This study revealed that people with unhealthy alcohol use may have problems not only with vitamin A deficiency, but also with a lack of RAR in the cells.

In our experiment, CD34^+^CD10^+^CD19^−^ cells from Alc did not differentiate into B cells. It has been reported that B cells in peripheral blood are reduced in Alc [29]. Furthermore, the number of B cells in the peripheral blood of patients with unhealthy alcohol use were lower than that of healthy donors in our study (Supplementary Figure S2). Alcohol causes deranged signal transduction, transcriptional and epigenomic changes, DNA damage, and changes in the fluidity and function of the plasma membrane. Therefore, it affects important aspects of stem cell biology, such as niche function, maintenance of potency, and differentiation [30]. In particular, interference with the sequential expression of transcription factors in early progenitor cells by alcohol exposure is a potential mechanism for the observed decrease in B cells, and alcohol affects the differentiation of progenitor B cells [31]. Furthermore, based on our study, we suggest that alcohol abstinence may improve the differentiation of progenitor B cells. This is the first report focusing on the mechanism by which abstinence restores the differentiation of CD34^+^CD10^+^CD19^−^ cells via the RA–RAR axis and restores host antibacterial resistance.

There was no difference in the number of CD34^+^CD10^+^CD19^−^ cells in the peripheral blood of HD and Alc. In our study, most of the CD34^+^CD10^+^CD19^−^ cells from HD differentiated into CD19^+^ cells, but not to CD3^+^, CD14^+^, or CD56^+^ cells under stimulation with RA. However, in the case of Alc, these cells were not differentiated into other cells after the same stimulation. Although CD34^+^CD10^+^CD19^−^ cells have the same number in the peripheral blood, they are progenitor cells that differentiate into other cells under other stimuli, and their number may be the same in different individuals. Further studies are required on the role and number of these cells.

HMGB1 are actively released from hepatocytes in response to alcohol consumption [32]. It has been reported that the amount of serum HMGB1 significantly increased in Alc with severe liver damage (serum AST > 80 U/L) However, there were only a few serum HMGB1 increases in Alc with mild liver damage (serum AST 40 U/L) [33]. In this study, only Alc with mild liver damage were enrolled. Therefore, HMGB1 from hepatocytes might not be significantly affected in serum. CD3^+^cells, CD14^+^cells, CD19^+^cells, and CD56^+^cells were isolated from peripheral blood of Alc, and these cells were cultured for 24 h, respectively. These cells produced few HMGB1 (Supplementary Figure S1). In our condition, CD34^+^CD10^+^CD19^−^ cells might serve as the major source of HMGB1.

HMGB1, LPS plus anti-ovalbumin (OVA) IgG/OVA immunocomplexes, anti-sheep erythrocyte IgG/erythrocyte immunocomplexes, and α1-acid glycoprotein have been reported to be specific inducers of M2bMφ/monocytes [34,35,36]. The role of HMGB1 has been demonstrated in the pathogenesis of sepsis and septic shock; it acts as a key “late-phase” inflammatory mediator [36]. Moreover, HMBG1 is known to upregulate the expression of miR-222 in some human cell lines [37]. In our recent studies, miR-222 was shown to stimulate M2bMφ/monocyte polarization in the mesenteric lymph nodes of γ-irradiated mice [38]. The expression of miR-222 in monocytes from Alc was detected. HMGB1 is actively secreted by innate immune cells, including monocytes, macrophages, and polymorphonuclear neutrophils in response to either LPS or TNF-α stimulation, and is released from damaged and tumor cells [39]. Once released, HMGB1 acts as a cytokine that exacerbates the inflammatory response by directly binding to TLR2 and TLR4 advanced glycation end products and syndecans [40]. In this study, the production of HMGB1 from CD34^+^CD10^+^CD19^−^ cells in AAlc was low compared to that in Alc. The reduction in the production of HMGB1 observed in our study might be enough to improve M2bMφ/monocyte polarization in Alc. In addition, long-term alcohol abstinence may be required to effectively reduce HMGB1 levels in the sera of Alc. Further studies are required in this regard.

## 5. Conclusions

CD34^+^CD10^+^CD19^−^ cells express RAR in HD, and RA stimulation dramatically promotes the differentiation of these cells into CD19^+^ cells. Because of the lack of expression of RAR, CD34^+^CD10^+^CD19^−^ cells from Alc fail to differentiate into CD19^+^ cells and these cells maintain the production of HMGB1, which induces M2b monocytes from resident monocytes. When the expression of RAR on CD34^+^CD10^+^CD19^−^ cells was restored by alcohol abstinence, these cells were differentiated into CD19^+^ cells. These cells did not produce HMGB1, and M2bMφ/monocytes, which reduce the host antibacterial resistance, were not induced.

## Figures and Tables

**Table 1:** Patients enrolled in this study.

	People with Unhealthy Alcohol Use	Healthy Donors
Total number of patients	18	11
Male/Female	13/5	9/2
Age (years), mean ± SD	55.2 ± 12.4	36.1 ± 7.4
Consumption of ethanol (g/day), mean ± SD	124.5 ± 63.6	6.6 ± 1.4
Duration of alcohol consumption (years), mean ± SD	26.7 ± 13.3	N/A
Total protein (g/dL), mean ± SD	7.2 ± 0.7	7.7 ± 0.4
Total bilirubin (mg/dL), mean ± SD	0.81 ± 0.55	0.46 ± 0.21
Aspartate aminotransferase (IU/mL), mean ± SD	41.2 ± 20.0	18.4 ± 4.8
Alanine transaminase (IU/mL), mean ± SD	29.9 ± 18.4	14.3 ± 5.4
γ-glutamyl transferase (IU/mL), mean ± SD	143.5 ± 106.2	34.5 ± 10.1
White blood cells (×10^3^/μL), mean ± SD	5.6 ± 2.1	7.0 ± 3.1
Platelets (×10^4^/μL), mean ± SD	24.7 ± 10.3	30.7 ± 10.9

## Supplementary Materials

The following supporting information can be downloaded at: https://www.mdpi.com/article/10.3390/cells11172703/s1, Figure S1: HMGB1 production from various cells in peripheral blood of patients with unhealthy alcohol use; Figure S2: Numbers of CD19^+^ cells in peripheral blood.

## References

[1] World Health Organization Global Status Report on Alcohol and Health 2018.

[2] Trevejo-Nunez G., Kolls J.K., de Wit M. (2015). Alcohol Use As a Risk Factor in Infections and Healing: A Clinician’s Perspective. Alcohol. Res..

[3] Tsuchimoto Y., Asai A., Tsuda Y., Ito I., Nishiguchi T., Garcia M.C., Suzuki S., Kobayashi M., Higuchi K., Suzuki F. (2015). M2b Monocytes Provoke Bacterial Pneumonia and Gut Bacteria-Associated Sepsis in Alcoholics. J. Immunol..

[4] Szabo G. (1999). Consequences of alcohol consumption on host defence. Alcohol. Alcohol..

[5] Siggins R.W., Melvan J.N., Welsh D.A., Bagby G.J., Nelson S., Zhang P. (2011). Alcohol suppresses the granulopoietic response to pulmonary Streptococcus pneumoniae infection with enhancement of STAT3 signaling. J. Immunol..

[6] Bagby G.J., Zhang P., Stoltz D.A., Nelson S. (1998). Suppression of the granulocyte colony-stimulating factor response to Escherichia coli challenge by alcohol intoxication. Alcohol. Clin. Exp. Res..

[7] Pascual M., Fernandez-Lizarbe S., Guerri C. (2011). Role of TLR4 in ethanol effects on innate and adaptive immune responses in peritoneal macrophages. Immunol. Cell Biol..

[8] Zisman D.A., Strieter R.M., Kunkel S.L., Tsai W.C., Wilkowski J.M., Bucknell K.A., Standiford T.J. (1998). Ethanol feeding impairs innate immunity and alters the expression of Th1- and Th2-phenotype cytokines in murine Klebsiella pneumonia. Alcohol. Clin. Exp. Res..

[9] Morland H., Johnsen J., Bjorneboe A., Bjorneboe G.E., Drevon C.A., Morland J., Morland B. (1988). Reduced IgG Fc-receptor-mediated phagocytosis in human monocytes isolated from alcoholics. Alcohol. Clin. Exp. Res..

[10] Bautista A.P. (2002). Chronic alcohol intoxication primes Kupffer cells and endothelial cells for enhanced CC-chemokine production and concomitantly suppresses phagocytosis and chemotaxis. Front. Biosci..

[11] Liu W., Li J., Tian W., Xu T., Zhang Z. (2011). Chronic alcohol consumption induces cardiac remodeling in mice from Th1 or Th2 background. Exp. Mol. Pathol..

[12] Laso F.J., Vaquero J.M., Almeida J., Marcos M., Orfao A. (2007). Chronic alcohol consumption is associated with changes in the distribution, immunophenotype, and the inflammatory cytokine secretion profile of circulating dendritic cells. Alcohol. Clin. Exp. Res..

[13] Pan H.N., Sun R., Jaruga B., Hong F., Kim W.H., Gao B. (2006). Chronic ethanol consumption inhibits hepatic natural killer cell activity and accelerates murine cytomegalovirus-induced hepatitis. Alcohol. Clin. Exp. Res..

[14] Starkenburg S., Munroe M.E., Waltenbaugh C. (2001). Early alteration in leukocyte populations and Th1/Th2 function in ethanol-consuming mice. Alcohol. Clin. Exp. Res..

[15] Ohama H., Asai A., Ito I., Suzuki S., Kobayashi M., Higuchi K., Suzuki F. (2015). M2b macrophage elimination and improved resistance of mice with chronic alcohol consumption to opportunistic infections. Am. J. Pathol..

[16] Joel M.D.M., Yuan J., Wang J., Yan Y., Qian H., Zhang X., Xu W., Mao F. (2019). MSC: Immunoregulatory effects, roles on neutrophils and evolving clinical potentials. Am. J. Transl. Res..

[17] Jiang Z., Han Y., Cao X. (2014). Induced pluripotent stem cell (iPSCs) and their application in immunotherapy. Cell Mol. Immunol..

[18] Honda Y., Kessoku T., Ogawa Y., Tomeno W., Imajo K., Fujita K., Yoneda M., Takizawa T., Saito S., Nagashima Y. (2017). Pemafibrate, a novel selective peroxisome proliferator-activated receptor alpha modulator, improves the pathogenesis in a rodent model of nonalcoholic steatohepatitis. Sci. Rep..

[19] Sanz E., Muñoz A.N., Monserrat J., Van-Den-Rym A., Escoll P., Ranz I., Alvarez-Mon M., de-la-Hera A. (2010). Ordering human CD34+CD10-CD19+ pre/pro-B-cell and CD19- common lymphoid progenitor stages in two pro-B-cell development pathways. Proc. Natl. Acad. Sci. USA.

[20] Bar-El Dadon S., Reifen R. (2017). Vitamin A and the epigenome. Crit. Rev. Food Sci. Nutr..

[21] Qiu J., Huang Y., Chen G., Chen Z., Tweardy D.J., Dong S. (2007). Aberrant chromatin remodeling by retinoic acid receptor alpha fusion proteins assessed at the single-cell level. Mol. Biol. Cell.

[22] Chen K., Ahmed S., Adeyi O., Dick J.E., Ghanekar A. (2012). Human solid tumor xenografts in immunodeficient mice are vulnerable to lymphomagenesis associated with Epstein-Barr virus. PLoS ONE.

[23] Shultz L.D., Lyons B.L., Burzenski L.M., Gott B., Chen X., Chaleff S., Kotb M., Gillies S.D., King M., Mangada J. (2005). Human lymphoid and myeloid cell development in NOD/LtSz-scid IL2R gamma null mice engrafted with mobilized human hemopoietic stem cells. J. Immunol..

[24] Agliano A., Martin-Padura I., Mancuso P., Marighetti P., Rabascio C., Pruneri G., Shultz L.D., Bertolini F. (2008). Human acute leukemia cells injected in NOD/LtSz-scid/IL-2Rgamma null mice generate a faster and more efficient disease compared to other NOD/scid-related strains. Int. J. Cancer.

[25] Hu Z., Van Rooijen N., Yang Y.G. (2011). Macrophages prevent human red blood cell reconstitution in immunodeficient mice. Blood.

[26] Asai A., Nakamura K., Kobayashi M., Herndon D.N., Suzuki F. (2012). CCL1 released from M2b macrophages is essentially required for the maintenance of their properties. J. Leukoc. Biol..

[27] Saha B., Bruneau J.C., Kodys K., Szabo G. (2015). Alcohol-induced miR-27a regulates differentiation and M2 macrophage polarization of normal human monocytes. J. Immunol..

[28] Wang L.X., Zhang S.X., Wu H.J., Rong X.L., Guo J. (2019). M2b macrophage polarization and its roles in diseases. J. Leukoc. Biol..

[29] Matos L.C., Batista P., Monteiro N., Ribeiro J., Cipriano M.A., Henriques P., Girão F., Carvalho A. (2013). Lymphocyte subsets in alcoholic liver disease. World J. Hepatol..

[30] Di Rocco G., Baldari S., Pani G., Toietta G. (2019). Stem cells under the influence of alcohol: Effects of ethanol consumption on stem/progenitor cells. Cell Mol. Life Sci..

[31] Wang H., Zhou H., Moscatello K.M., Dixon C., Brunson L.E., Chervenak R., Chervenak D.C., Zhao X., Wolcott R.M. (2006). In utero exposure to alcohol alters cell fate decisions by hematopoietic progenitors in the bone marrow of offspring mice during neonatal development. Cell Immunol..

[32] Gaskell H., Ge X., Nieto N. (2018). High-Mobility Group Box-1 and Liver Disease. Hepatol. Commun..

[33] Vannier A.G.L., Wardwell B., Fomin V., PeBenito A., Wolczynski N., Piaker S., Kedrin D., Chung R.T., Schaefer E., Goodman R. (2021). Serum HMGB1 associates with liver disease and predicts readmission and mortality in patients with alcohol use disorder. Alcohol.

[34] Mantovani A., Sozzani S., Locati M., Allavena P., Sica A. (2002). Macrophage polarization: Tumor-associated macrophages as a paradigm for polarized M2 mononuclear phagocytes. Trends Immunol..

[35] Nakamura K., Ito I., Kobayashi M., Herndon D.N., Suzuki F. (2015). Orosomucoid 1 drives opportunistic infections through the polarization of monocytes to the M2b phenotype. Cytokine.

[36] Wang H., Yang H., Czura C.J., Sama A.E., Tracey K.J. (2001). HMGB1 as a late mediator of lethal systemic inflammation. Am. J. Respir. Crit. Care Med..

[37] Mari E., Zicari A., Fico F., Massimi I., Martina L., Mardente S. (2016). Action of HMGB1 on miR-221/222 cluster in neuroblastoma cell lines. Oncol. Lett..

[38] Suzuki F., Loucas B.D., Ito I., Asai A., Suzuki S., Kobayashi M. (2018). Survival of Mice with Gastrointestinal Acute Radiation Syndrome through Control of Bacterial Translocation. J. Immunol..

[39] He S., Cheng J., Sun L., Wang Y., Wang C., Liu X., Zhang Z., Zhao M., Luo Y., Tian L. (2018). HMGB1 released by irradiated tumor cells promotes living tumor cell proliferation via paracrine effect. Cell Death Dis..

[40] Andersson U., Tracey K.J. (2011). HMGB1 is a therapeutic target for sterile inflammation and infection. Annu. Rev. Immunol..

