# Supplementary material for: CD34+CD10+CD19− Cells in Patients with Unhealthy Alcohol Use Stimulate the M2b Monocyte Polarization
Source: Cells. 2022 Aug 30;11(17):2703. doi: 10.3390/cells11172703 (PMC9454773; doi:10.3390/cells11172703)
Supplement: Supplementary file 1 [file cells-11-02703-s001.zip › cells-1829808-supplementary.pdf]

Supplementary Materials

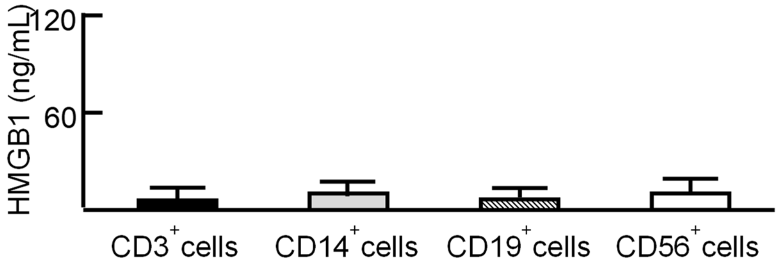

Figure S1. $CD3^+$ cells, $CD14^+$ cells, $CD19^+$ cells and $CD56^+$ cells were isolated from PBMC of patients with unhealthy alcohol use by magnetic isolation methods, respectively. These cells (5×10^5 cells /mL) were cultured for 24 h. The culture fluids were obtained and HMGB1 was assayed for ELISA.

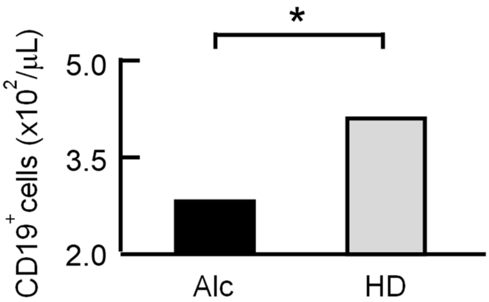

Figure S2. The number of $CD19^+$ cells in peripheral blood was compared between patients with unhealthy alcohol use and health donors.
